# Challenges Faced by Prehospital Emergency Physicians Providing Emergency Care to Patients with Advanced Incurable Diseases

**DOI:** 10.1155/2019/3456471

**Published:** 2019-11-26

**Authors:** Anne Kamphausen, Hanna Roese, Karin Oechsle, Malte Issleib, Christian Zöllner, Carsten Bokemeyer, Anneke Ullrich

**Affiliations:** ^1^Department of Anesthesiology, University Medical Center Hamburg-Eppendorf, Hamburg, Germany; ^2^Palliative Care Unit, Department of Oncology, Hematology, and Bone Marrow Transplantation, University Medical Center Hamburg-Eppendorf, Hamburg, Germany

## Abstract

**Introduction:**

The aim of our study was to investigate challenges faced by emergency physicians (EPs) who provide prehospital emergency care to patients with advanced incurable diseases and family caregivers in their familiar home environment.

**Methods:**

Qualitative study using semistructured interviews with open-ended questions to collect data from 24 EPs. Data were analyzed using qualitative content analysis.

**Results:**

We identified nine categories of challenges: structural conditions of prehospital emergency care, medical documentation and orders, finding optimal patient-centered therapy, uncertainty about legal consequences, challenges at the individual (EP) level, challenges at the emergency team level, family caregiver's emotions, coping and understanding of patient's illness, patient's wishes, coping and understanding of patient's illness, and social, cultural, and religious background of patients and families. EPs strengthened that the integrations of specialized prehospital palliative care services improved emergency care by providing resources to patients and family caregivers, enhancing the quality and availability of medical documentation and accessibility of aftercare in emergencies. Areas of improvement that were identified were to promote emergency physicians' knowledge and skills in palliative care, communication, and family caregiver support by education and training. Furthermore, structures for better care on-site, thorough medical documentation, and specialized palliative care emergency facilities in hospital and prehospital care were requested.

**Conclusion:**

Prehospital emergency care in patients with advanced incurable diseases in their familiar home environment may be improved by training EPs in palliative care, communication, and caregiver support competences. Results underline the importance of collaborative specialized palliative care and prehospital emergency care.

## 1. Introduction

Palliative care intends to improve the quality of life of patients suffering from advanced incurable, life-limiting diseases and by enabling them to live a dignified life until patients' death in the desired environment. At end of life, the majority of patients prefer to receive care and die in their familiar home environment—in Germany, this applies to approximately two-thirds of all patients with advanced cancer [1]. Patients with advanced incurable diseases and family caregivers can face diverse sudden and sometimes acute life-threatening problems in their familiar home environment, such as dyspnea, convulsive attacks, pain exacerbation, or severe anxiety, which may lead to emergency calls and subsequent hospital admissions [2].

Previous studies have shown that the number of prehospital emergency care visits ranges from 2–10% in patients with advanced incurable diseases in their familiar home environment [3–6]. Therefore, every prehospital provider can be confronted with patients at an advanced stage of disease who need emergency medical care [6]. The majority of prehospital emergency calls and emergency department visits of patients with advanced incurable diseases are caused by shortness of breath, pain, neurological disorders, family caregiver exhaustion [7–9], by disease-related anxiety, or feelings of safety and familiarity with the hospital setting [9, 10] and are dependent upon local community-based healthcare structures and population areas [11]. Additionally, studies showed that some emergency department visits might be avoidable and are related to some such as pain medication refills or constipation [12]. The use of emergency medicine kits in home hospice patients seems to be helpful and might provide a timely symptom relief and may diminish the need for prehospital emergency care [13, 14]. Nevertheless, there are still unavoidable emergency department visits related to infections or neurologic events.

To improve home care of patients with advanced incurable diseases and with complex symptom burden, specialized prehospital palliative care services were established in Germany in 2007 [15]. These services are at least biprofessional, include general practitioners or family physicians with specific board qualification in palliative care and nurses, and are 24 hours available. In addition to medical and patient care in the home environment, their responsibilities include the coordination of care and the provision of psychosocial support to both patients and family caregivers [15, 16]. Prior studies indicated that the involvement of palliative care can reduce prehospital emergency care in patients with advanced incurable diseases in their familiar home environment [7, 9, 12, 16–20] and avoid hospital admissions [7, 12, 18]. A German study showed that in approximately 97% of patients cared for by specialized prehospital palliative care services, no prehospital emergency care was necessary and hospital admissions were avoided in 80% of the cases [16]. Nevertheless, these services are not available nationwide and optimal patient care remains insufficient [21].

From the point of view of specialized prehospital palliative care services, several difficulties in emergency care for patients with advanced incurable diseases in their familiar home environment have been identified: inadequate end-of-life discussions, missing documentation on treatment goals in emergency situations, e.g., “do not resuscitate orders,” or explicit wishes of patients or family caregivers not to be admitted to the hospital [22].

In addition to these findings, several problems in the treatment of patients with advanced incurable diseases in the emergency department (ED) have been identified [23–27]: working conditions such as fast-paced work environment, quickly needed treatment decisions, challenges associated with cultural differences, as well as a lack of patient-physician relationship. Inadequate skills in the treatment and communication of palliative care patients can entail challenges for emergency physicians (EPs) in the emergency department [28]. Even though knowledge about palliative care is considered to be an important competency in inpatient emergency care [28, 29], the understanding and knowledge regarding palliative care is often limited in emergency department EPs [30, 31]. Structured training of EPs working in the ED in palliative care improves their medical skills in palliative care, which can enhance patient care in emergency departments [28]. Challenging aspects of emergency care for patients with advanced incurable diseases in the emergency department might also occur in prehospital emergency care, and specific requirements of prehospital emergency care are likely to influence both the extent and quality of challenges faced by prehospital emergency physicians (EPs).

Therefore, our study aimed to explore challenges prehospital EPs face during prehospital emergency care for patients with advanced incurable diseases in their familiar home environment. This included an evaluation of prehospital emergency physicians' understanding of palliative care, challenges experienced during prehospital emergency care for patients with advanced incurable diseases in their familiar home environment, and suggestions of potential improvements.

## 2. Methods

### 2.1. Study Design

An explorative qualitative design using semistructured interviews was used. The study focused on challenges prehospital EPs experience when providing patients with advanced incurable diseases and family caregivers with prehospital emergency care in their familiar home environment. End-of-life care was included as a specific part of palliative care. Since general prehospital palliative care was not defined by specific care structures in Germany at that time, the term “patients with advanced incurable disease in their familiar home environment” was left partly undefined intentionally to include challenges caused by missing definitions and structures in this context.

This study was conducted at a department of anesthesia at a university medical center in Hamburg, Germany. At the time of the study, there were 32 emergency physicians in the anesthesiology department which provided prehospital emergency care at a fire and rescue station in Hamburg. All participants had at least 6 months of professional experience in prehospital emergency care. Given emergency physicians' written informed consent, the interviewer (HR), who had no clinical or personal relationship with the participants, conducted semistructured face-to-face interviews, which were audiotaped, transcribed verbatim, and anonymized. Basic data on sociodemographic characteristics and emergency physicians' professional experience were also inquired.

The study has been approved by the local ethics committee of Hamburg, Germany (PV5217).

### 2.2. Data Collection

Interviews were conducted in the premises of the University Medical Center Hamburg-Eppendorf between November 2016 and March 2017. A structuring interview guide with open-ended questions was used, which had been developed by an interdisciplinary research group. The guide began with questions regarding basic understanding of palliative care, with a focus on challenging factors of prehospital emergency care in patients with advanced incurable diseases in their familiar home environment. EPs were encouraged to recall about experiences they made with and without the involvement of specialized prehospital palliative care services. Written prompts with the following keywords were used to help the EPs to structure their thoughts regarding potential challenges: “structures,” “processes,” “outcomes,” “team-based,” “individual,” and “social.” Interviews were closed with questions regarding the improvement of prehospital emergency care in patients with advanced incurable diseases in their familiar home environment.

### 2.3. Data Analyses

The interviews were analyzed using qualitative content analysis with an inductive approach according to Mayring [32, 33]. The first five interviews were independently analyzed by AK and HR (medical researchers), who first assigned suitable codes to relevant text passages of the interview transcripts. To check generated codes, coded interview transcripts were discussed in several meetings within the research team including researchers with expertise in qualitative research. In the course of this process, all codes were open to adaptation. After development of the final coding scheme, all interviews were analyzed by AK and HR using the data analysis program MAXQDA. Differences between encoders were resolved by verbal discussions.

We used the Consolidated Criteria for Reporting Qualitative Studies (COREQ) framework to report on the design, conduct analysis, and findings of our study [34].

## 3. Results

### 3.1. Characteristics of the EPs

We interviewed 24 out of 32 EPs with the number of interviews and sampling basing on the principles of maximum variation regarding gender and experience in prehospital emergency care. Ten EPs were female and 14 male with a mean professional experience in anaesthesiology of 8.1 years (range, 4.5–14). Six participants had professional experience in emergency medicine of less than 3 years, 12 of 4–6 years, and six of 7 years or more. One EP had obtained specific board qualification in palliative care. The interviews lasted 48 minutes (range, 29–70) on average.

### 3.2. Understanding of Palliative Care

When describing their conception of palliative care, all EPs unanimously pointed to the care of terminally ill or dying patients, alleviation of suffering, medical symptom control, and improvement of quality of life. Knowledge about palliative care varied widely. EPs unanimously reported that prehospital emergency care patients with advanced incurable diseases in their familiar home environment mostly occurred in cases of insufficient symptom control with exacerbation of pain or dyspnea being mentioned most frequently. Secondly, high family caregiver burden in terms of anxiety, poor preparedness to respond to emergency situations, or general helplessness were identified to provoke emergency calls.

### 3.3. Challenges in Prehospital Emergency Care

Interviews revealed nine categories of challenges that EPs faced when providing prehospital emergency care to patients with advanced incurable diseases in their familiar home environment.

1. Structural conditions of prehospital emergency care
2. Medical documentation and orders
3. Finding optimal and patient-centered therapy
4. Uncertainty about legal consequences
5. Challenges at the individual (EP) level
6. Challenges at the emergency team level
7. Family caregiver's emotions, coping, and understanding of patient's illness
8. Patient's wishes, coping, and understanding of patient's illness
9. Social, cultural, and religious background of patients and families

Main categories, subcategories with definitions, and exemplary quotes are listed in Table 1.

#### 3.3.1. Structural Conditions of Prehospital Emergency Care

EPs unanimously reported that prehospital emergency care is subjected to time limits, which may contribute to subsequent hospitalization of the patient. EPs often could not dedicate the time needed for end-of-life discussions including prognosis and therapy goals. Furthermore, EPs described inadequate medical equipment, e.g., specific medicine for palliative—not emergency—care, to cause problems.

#### 3.3.2. Medical Documentation and Orders

In all interviews, EPs described that contradictory and missing documents about the patient's illness, past medical treatment, and emergency orders cause problems, particularly in high emergencies. In many cases, no advanced directives had been prepared but were considered highly important if the patient was no longer capable of expressing his or her own will. In addition to missing documentation, ambiguous or contradictory wording was described as challenging for treatment decisions.

#### 3.3.3. Finding Optimal and Patient-Centered Therapy

EPs considered it demanding to find optimal and patient-centered therapy in the prehospital emergencies due to restricted equipment and a large variety of patients' wishes regarding therapy. This was particularly relevant in the presence of insufficient medical documentation and orders. Lacking knowledge of treatment schemes and options in palliative care was perceived to complicate decision-making.

#### 3.3.4. Uncertainty about Legal Consequences

Most EPs reported to fear potential legal consequences of treatment, in particular when administering high-dose opioids. Prehospital emergency care was claimed to often result in hospitalization of patients because EPs were uncertain about legal issues and how to assure patient safety at home without having the possibility to observe treatment outcomes.

#### 3.3.5. Challenges at the Individual (EP) Level

Even experienced EPs described an impact on personal needs, emotional reactions to the situation faced (e.g., sadness), and prior family or work experiences with palliative care situations. Showing empathy to patients and family caregivers was considered highly important among EPs, but some pointed out that empathetic behavior often has to stand back in general prehospital emergency care, and thus can challenge EPs when required. Lack of professional experience and knowledge in palliative care was mentioned to lead to challenges, especially for younger EPs.

#### 3.3.6. Challenges at the Emergency Team Level

EPs reported that challenges could arise from diverging perceptions and reactions regarding the care for a terminally ill or imminently dying patient within the emergency team (physicians and paramedics). Decision-making and treatment plans are structured by hierarchies but might be less effective when team members disagree. Additionally, lacking empathy of colleagues was reported to have led to team conflicts in several cases.

#### 3.3.7. Family Caregiver's Emotions, Coping, and Understanding of Patient's Illness

Almost all EPs described burden of family caregivers (e.g., anxiety and exhaustion), limited understanding of patient's illness, avoiding coping strategies, and strong emotional involvement as challenging factors in prehospital emergency care. EPs underlined that communicating with affected family caregivers requires time and specific skills, which are both limited in prehospital emergency care.

#### 3.3.8. Patient's Wishes, Coping, and Understanding of Patient's Illness

EPs mentioned patients' limited understanding of illness, anxiety, and avoiding coping strategies, which often result in difficult treatment and end-of-life care discussions, to be challenging. Furthermore, consideration of patients' wishes regarding emergency care is often difficult in prehospital emergency care due to a deteriorated clinical status (e.g., unconsciousness and weakness) or conflicting caregiver needs.

#### 3.3.9. Social, Cultural, and Religious Background of Patients and Families

Cultural norms or religious beliefs regarding life and death as well as expectations towards the treatment of terminally ill or dying patients were mentioned to challenge EPs. Limited health literacy of patients or families was perceived as a general problem in prehospital emergency care, which even aggravated in patients with advanced incurable diseases.

### 3.4. Perceived Impact of Specialized Prehospital Palliative Care Services

All interviewed EPs agreed that emergency care in patients with advanced incurable diseases in their familiar home environment substantially improves if a specialized prehospital palliative care service is involved as follows:

#### 3.4.1. Patients' and Family Caregivers' Preparedness

EPs felt that patients and family caregivers had often gained a deeper understanding of the patient's clinical status, were better prepared for emergencies, and overall caregiver burden seemed reduced, which promoted patient-centered treatment decisions.

#### 3.4.2. Leaving Patients Safely at Home

The availability of aftercare in terms of rapid response by specialized prehospital palliative care services was experienced to prevent subsequent hospital admissions, as specialized prehospital palliative care services could monitor treatment outcomes and EPs knew patients to be cared in a safe environment.

#### 3.4.3. Quality of Medical Documentation

EPs reported documentation about patients' medical/treatment history and emergency orders was valuable and easily available, which contributed to a rapid and adequate response to the emergencies. Advanced directives were prepared more often when specialized prehospital palliative care services were involved and EPs felt relieved that they knew the patients' wishes in the run-up to treatment decisions.

### 3.5. Improvement Suggestions

EPs proposed improvements of prehospital emergency care for patients with advanced incurable diseases in their familiar home environment (Table 2).

#### 3.5.1. Education and Training in Palliative Care

Lacking knowledge and skills in palliative care was stressed to complicate prehospital emergency care in patients with advanced incurable diseases in their familiar home environment. Integrated structured training in EPs education and specific workshops or practical training (e.g., attending a specialized palliative care unit) could enhance optimal care from EPs point of view. Additionally, EPs described a need for transparent information about existing in hospital and prehospital palliative care structures, as well as their indication and utilization.

#### 3.5.2. Structural Modifications

EPs suggested standardized emergency orders including information about illness, past medical treatment, and drug therapy. Furthermore, they proposed the establishment of a “rapid response team” for patients with advanced incurable disease who were not receiving palliative care by a specialized prehospital palliative care service and a regional advisory hotline (specialized prehospital palliative care service) for EPs. To avoid stressful transition via hospital emergency wards, EPs thought of direct admissions to specialized palliative care facilities in hospitals.

## 4. Discussion

This qualitative study describes various challenges EPs face when providing prehospital emergency care to patients with advanced incurable diseases in their familiar home environment. Structural conditions, barriers for optimal patient-centered care, lacking medical orders, legal uncertainties, lack of palliative care knowledge, and personal experiences were described to cause demanding situations. Additionally, patients' and particularly family caregivers' avoiding coping strategies and limited understanding of patient's illness, and exhaustion were identified as potential complicating factors.

EPs pointed out various challenges similar to those experienced by providers in hospitals' emergency departments, e.g., differing cultural norms, spiritual beliefs, and limited health literacy [23–27]. Furthermore, emergency department physicians have described that many patients were not aware of their clinical status, often due to lack of primary care, unreasonable expectations, or limited health literacy [23], which was similar to the statements of the EPs in our study. They also reported an oftentimes limited understanding of patient's illness, which could be enhanced by the involvement of specialized prehospital palliative care services. Challenges associated with working conditions, such as fast-paced work environment, the need for rapid treatment decisions, as well as a lack of patient-physician relationship seen in in hospital emergency care [23–27], were also repeatedly mentioned by EPs in our study. Other studies had observed significantly longer mean durations of emergency care visits in patients with advanced incurable diseases in their familiar home environment, which might result from emergency physicians' doubts to leave the patients in an insecure situation at home [35]. In our study, EPs indicated fear of legal consequences. Furthermore, our study revealed that lacking medical equipment, e.g., specific medication for palliative—not emergency—care, also causes challenging situations. In literature, some studies focussing on the use of emergency medicine kits in home hospice patients are indicating that these medications kits could be helpful to provide timely symptom relief and may diminish the need for prehospital emergency care [13, 14]. These findings add some new aspects to improve prehospital emergency care in patients with advanced incurable diseases in their familiar home environment.

In our study, EPs emphasized their feeling that prehospital emergency care is relevantly improved if specialized prehospital palliative care services are involved in the care of patients with advanced incurable diseases in their familiar home environment, e.g., to leave patients in safe follow-up care at home. Our findings indicate the value of specialized prehospital palliative care services for quality prehospital emergency care in patients with advanced incurable diseases in their familiar home environment, which is underlined by studies reporting that involvement of such specialized services can reduce prehospital emergency care and avoid hospital admission [7, 9, 12, 16–20]. Studies focussing on the perspective of specialized prehospital palliative care services have pointed out that cooperation between prehospital emergency care and prehospital palliative care should be strengthened and requires joint future approaches for collaboration [22, 36]. This was also observed within the emergency physicians' perspective in our study, which should motivate politicians and healthcare systems to facilitate structural modifications. Moreover, literature emphasizes that integration of general practitioners and family physicians can decrease the number of hospitalization by the increase of continuous care and may strengthen a continuous care in the home environment [37].

Emergency physicians pointed out that challenges could arise from diverging perceptions and reactions regarding the care for a terminally ill or imminently dying patient within the emergency team (physicians and paramedics). Decision-making and treatment plans are structured by hierarchies but might be less effective when team members disagree. Additionally, lacking empathy of colleagues was reported to have led to team conflicts in several cases. A study could show that adverse events can be attributed to a lack of teamwork. Effective teamwork may improve the quality of care [38]. Our findings emphasize the importance of team training concepts with focus on communication, coordination, and leadership that support effective teamwork.

Prior studies showed that EPs had a significantly lower knowledge about pain therapy in cancer patients than palliative care physicians [39]. Furthermore, education on decisions in end-of-life therapy and legal consequences of patients' wishes were considered relevant for EPs [27, 40]. Lacking knowledge about palliative care as well as limited professional experience in prehospital emergency care for patients with advanced incurable diseases in their familiar environment care was reported throughout our study. The need for structurally integrated education and training of palliative care for EPs became evident. Previous studies have shown that structured training in palliative care, e.g., with case-based simulation interventions, can improve emergency physicians' medical skills and enhance inpatient emergency care [27, 41]. It has even been suggested that hospice and palliative care might be a new “subspecialty of emergency medicine” [41]. Some first examples for recommendations for palliative care in emergency care have already been published [6, 42, 43]; however, they are currently not included sufficiently in standard recommendations for daily clinical practice.

Limitations of our study include the explorative character and the selectivity of the study cohort. The study cohort comprised only EPs of one single fire and rescue station. However, this station covers different population areas representing the entire social and cultural range of a large urban city. All interviews were performed on a purely voluntary basis. Therefore, we could not exclude social desirability as well as an experiential response. The semistructured approach secured the relevant content and left enough time for the interviewed EPs to give a broad range of opinions of prehospital emergency care for patients with advanced incurable diseases in their familiar environment.

## 5. Conclusion

In conclusion, our study indicates many challenges faced by prehospital EPs providing emergency care to patients with advanced incurable diseases and family caregivers in their home environment. From the emergency physicians' perspective, the integration of specialized prehospital palliative care services seems to improve handling of such emergencies and helps to avoid subsequent unwanted emergency department admissions. Improved structures for integration of palliative care in prehospital emergency medicine and connecting specialized palliative care and emergency care are needed. Knowledge about palliative care, communication, and family caregiver support should be part of emergency physicians' education and training.

## Figures and Tables

**Table 1:** Challenges in prehospital emergency care in patients with advanced incurable disease in their familiar home environment.

Categories and subcategories	Definitions and examples
*Category 1*	Structural conditions of prehospital emergency care
1.1. Limited time	Complex symptoms and situations in prehospital emergencies in patients with advanced incurable disease require more time than available in an emergency system, which is focused on fast acting rescue teams “[…] I do not have the time to take palliative care of a patient at home or even develop a palliative concept […] I have to decide whether the patient can stay at home or needs to be taken to a hospital” (3, 14)
1.2. Lacking medicine and medical equipment	Medical supplies are limited and do not necessarily include the needed items in a palliative situation “We have restricted varieties of medication and medical equipment, therefore my options are very limited […]” (4, 51)

*Category 2*	Medical documentation and orders
2.1. Documentation of illness, past medical treatment and drug therapy	Often there is no precise material about the past medical history and treatment plans at the patients' home “A large problem is the documentation. Standard reply: It's at my general practitioner's office But as an emergency physician I'm not there during the general practitioner's working hours, instead at night, on weekends” (9, 55)
2.2. Advanced directives	Advances directives' importance if the patient is no longer capable of expressing an own will “It's difficult for me when I only have the assumed will I'm told by relatives […] and nothing written. I'm then leaving an end-of-life patient that's most likely to die […] but personally, I've got nothing but the assumed will from people, mostly even without a healthcare proxy” (19, 52)

*Category 3*	Finding optimal and patient-centered therapy
	Restricted resources and a large variety of patient wishes regarding therapy and their impact on medical emergency decision-making “Those situations present a conflict on how much to do, how much I can do and still let the patient remain at home” (6, 2)

*Category 4*	Uncertainty about legal consequences
	Decision-making regarding treatment/stay at home and the impact of possible legal consequences “[…] with pain medication there's always the danger of shortening it [life]. I give him pain medication and he gets respiratory insufficient. What are the legal consequences?” (13, 25)

*Category 5*	Challenges at the individual (EP) level
5.1. Personal needs and emotions	Personal needs and emotions expressed by the interviewed EPs when facing patients with advanced incurable diseases “If I leave patients at home I need to know that all people are informed about things to come, can cope with the situation and that suffering is bearable” (2, 2)
5.2. Experiences with palliative care situations	Personal and work experience with patients with advanced incurable disease and treatment “But to deal with this hopeless situation, it is hard to accept that this is not a personal failure and it is hard to accept that I cannot longer help this patient […]” (7)
5.3. Lacking palliative care knowledge and expertise	Lacking palliative care knowledge due to lack of education and training “We or some emergency physicians and paramedics aren't educated and have no specific knowledge on how to cope with those patients and what to do” (20, 66)
5.4. Empathetic behavior	Challenges for empathetic behavior for EPs in the special case of emergencies in patients with advanced incurable disease in their home environment “However, I think most important are empathy skills, to get involved with the patient and act correctly and adequately in the situation […] this becomes a problem if such skills are lacking” (16, 51)

*Category 6*	Challenges at the emergency team level
6.1. Hierarchies	Impact of the emergency team hierarchy on decision-making and treatment plans “Disagreements in the team. […] we have a clear hierarchy and the emergency physician is in charge of making the decision what to do and when to stop. When you're unsure, this spreads over the team and the leading position can change” (2, 80)
6.2. Coping with palliative care situations	Different concepts of coping with a patient with advanced incurable disease within the team “I might be responsible but the whole team has to cope when I decide not to resuscitate. You need to talk about it beforehand. […] especially in palliative emergency settings the team is vital” (16, 47)

*Category 7*	Family caregivers' emotions, coping, and understanding of patient's illness
7.1. Caregivers' understanding of the patient's clinical status	Perception and expectations towards the clinical situation in patients with advanced incurable disease as seen by family caregivers “I believe it is important for relatives to know and to be incorporated, but that's something I experience isn't all too apparent in communication. […] because they sometimes have completely different expectations, I believe you must have honest conversations on how it's going to be” (6, 2)
7.2. Family caregivers' emotions and coping with end-of-life situations	Emotional responses and (dysfunctional) coping strategies of family caregivers in end-of-life situations “Most times we're not being called for the patient but because the relatives don't know how to cope with the situation” (1, 26)

*Category 8*	Patient's wishes, coping, and understanding of patient's illness
8.1. Patient's coping and understanding of patient's illness	Varying knowledge and coping strategies from patients in palliative end-of-life situations influences the treatment in emergency settings “Sometimes patients aren't informed about all the options they have. They're sometimes discharged or at least that's what they say, without knowing what to do in those (emergency) situations” (6, 2)
8.2. Patients' wishes	Patients' wishes and expectations at end-of-life and influence on treatment options “I experience quite a lot that I'm unsure of what to do with a patient and that's usually because of a discrepancy between what the patient wants on one hand, which is not to be taken to a hospital, the wish to die at home, nursing home or wherever. Quiet without suffering. And what relatives dictate on the other hand” (10, 36)

*Category 9*	Social, cultural, and religious background of patients and families
9.1. Cultural/religious beliefs	Belief systems and cultural settings have an impact on the options and wishes in emergency care in patients with advanced incurable disease “In some cultures, it is common that more and more people enter the room every minute. It makes it impossible for me to find out what's the patient's problem, that's stressful” (9, 78)
9.2. Health literacy	Different socioeconomic statuses and knowledge about options in the healthcare system affect the medical background “Patients with higher living standards have usually more specific wishes than patients who belong to lower social classes, hence have more often a specialized prehospital palliative care service involved” (1, 74)

Abbreviations: EP, emergency physician.

**Table 2:** Improvement suggestions for prehospital emergency care in patients with advanced incurable disease.

Categories and subcategories	Definitions and examples
*Category 1*	Structural modifications
1.1. Emergency system structure	Structures for better care on-site and further support “It [the emergency system] is not prepared for it. […] and the resources because our vehicles aren't equipped for it” (1, 103)
1.2. Documentation	Thorough documentation to help clarify the situation and justify therapy in patients with advanced incurable diseases “Information needs to be better prepped and communicated. Like a map or something. There's always an info sheet. But that's never on palliative care. Always general info like the medication chart. But never a palliative care concept. That would be beautiful, but it hardly ever happens” (2, 90)
1.3. Specialized prehospital palliative care services accessibility	A wider range of specialized palliative care emergency services for focused treatment “The only preferable option, if palliative care facilities were to be more centered for those patients. For strategies with a primary acute palliative care team that's called instead of an emergency physician” (18, 106)

*Category 2*	Education and training
2.1. Adaptations in EP qualification	Palliative care as an addition to the standard emergency care curriculum “It's not mentioned on the emergency physician course. How to treat palliative care patients is not ranked very high in the emergency medicine educational world, but trauma, resuscitation, cardiac insufficiency and coronary heart disease” (21, 59)
2.2. Further practical training/experience in palliative care	Practical experience in a specialized palliative care unit and further training in applied palliative care “If you could organize for us emergency physicians to join the palliative care unit. For more information and a certain practice. Those two things would help, would help me” (13, 66)
2.3. Further qualifications informing on palliative care and facilities	Workshops and widespread information about palliative care and available structures, their indication, and utilization “To get to know more about prehospital palliative care, whether there are options for a quick organisation of such care” (5, 80)

Abbreviations: EP, emergency physician.

## Data Availability

The authors have full control over the primary data. The data analyzed in this study are housed at the Palliative Care Unit, Department of Oncology, Hematology and BMT, University Medical Center Hamburg-Eppendorf, Martinistrasse 52, 20246 Hamburg, Germany. According to the ethical committee approval, this dataset is subject to ethical restrictions and local data protection regulations regarding qualitative raw data, since participant privacy could be compromised. Participants did not consent to have their full transcripts made available for third parties. All relevant data for the conclusions are presented in the manuscript.

## Abbreviations

COREQ: Consolidated criteria for reporting qualitative studies
EP: Emergency physician.
